# Axial variation in conduit dimensions in angiosperm leaves and conifer needles — a review

**DOI:** 10.3389/fpls.2026.1873867

**Published:** 2026-07-01

**Authors:** Roman Gebauer

**Affiliations:** Department of Forest Botany, Dendrology and Geobiocoenology, Faculty of Forestry and Wood Technology, Mendel University in Brno, Brno, Czechia

**Keywords:** allometric scaling, axial variation, leaf hydraulics, multipoint vascular analysis, xylem anatomy

## Abstract

The tip-to-base widening of xylem conduits is a well-documented feature of plant hydraulic architecture at the level of stems and branches. This anatomical pattern, predicted by hydraulic optimization theory (WBE model) minimizes the increase in hydraulic resistance associated with increasing conductive path length. Because the WBE model is not directly applicable to leaves, which function as near two-dimensional, laterally leaky vascular networks, an extended WBE framework was later developed to account for additional aspects of leaf vascular organization. This review focuses on only one component of that broader design – leaf level tip-to-base conduit widening. To date, only seven studies have directly quantified tip-to-base changes in conduit diameter within leaves or needles. Unlike stem, the exponent characterizing conduit widening in leaves is not universal across vascular plant lineages, likely reflecting differences in the geometric and functional constraints governing leaf vascular networks. An additional five studies have addressed spatial variation across the leaf vein hierarchy or scale petiole conduit diameter with leaf size. This limits our understanding of the functional significance of leaf-level xylem structural changes and how this differs systematically among lineages. Hence, this review emphasizes the need to unify methodologies for analyzing leaf xylem structure through the inclusion of hydraulic path length measurements. Nevertheless, advancing the field requires not only incorporating leaf-level tip-to-base conduit widening, but also adopting integrative sampling strategies that capture multiple dimensions of leaf hydraulic organisation within plant water transport frameworks.

## Introduction

The transport of water from soil to the atmosphere through plants is one of the central physiological processes enabling terrestrial life. This transport is mediated primarily by the xylem, a highly specialized vascular tissue composed of dead, hollow conduits. The geometry of these conduits (i.e. diameter, length, connectivity, and spatial arrangement) exerts strong control over the hydraulic resistance of the transport pathway (Sack and Holbrook, 2006). Xylem conduit diameter, length, and pit characteristics scale axially in trees in a coordinated manner that maintains a balance between lumen and end-wall resistivity, thereby optimizing hydraulic efficiency and preserving stable hydraulic function with increasing plant height (Schulte, 2012; Lazzarin et al., 2016; Zambonini et al., 2024; Kreinert et al., 2025; Held et al., 2026). Because the hydraulic conductance of capillary-like tubes scales with the fourth power of conduit radius (Hagen–Poiseuille law), even small changes in conduit diameter can strongly influence water transport efficiency (Tyree and Zimmermann, 2002). Xylem structure therefore underpins whole-plant water transport, linking hydraulic conductance to stomatal control, carbon assimilation, and plant persistence under drought (Venturas et al., 2017).

Understanding how xylem conduit dimensions vary along the soil–plant–atmosphere continuum has therefore been a longstanding objective of plant anatomy and physiology. Early anatomical work already documented that xylem conduits are narrow at distal positions along the transport pathway and widen toward the plant base (Grew, 1682 in Olson et al., 2021). These empirical observations raised fundamental questions about the functional significance of conduit widening and its role in compensating for increasing transport distance as plants grow taller. A major conceptual advance in this field came with the formulation of the West–Brown–Enquist (WBE) model, which provided a theoretical framework linking xylem anatomy to hydraulic optimization (West et al., 1999). The WBE model predicts that conduit diameter increases basipetally following a power-law relationship with distance from the apex, with an exponent of 0.25. This degree of widening is sufficient to offset increasing path length and minimizing hydraulic resistance. Empirical studies across angiosperms, gymnosperms, and non-seed plants confirm that stem and branch xylem generally follow this prediction, with a typical scaling exponent of approximately b = 0.2, although values can range from 0.1 to 0.4 depending on the species (e.g. Anfodillo et al., 2006; Petit et al., 2014; Rosell and Olson, 2014; Lazzarin et al., 2016; Zambonini et al., 2024; Petit, 2024; Kreinert et al., 2025; Held et al., 2026). This pattern has been interpreted as evidence for adaptive optimization balancing hydraulic efficiency, safety from embolism, and construction costs (Mencuccini et al., 2007). Nevertheless, recent perspectives suggest that conduit widening alone cannot fully explain the maintenance of hydraulic function with increasing height. It has been proposed that trees may sustain constant leaf-specific conductance through increased xylem permeability, although these adjustments may increase embolism risk in taller plants (Prendin et al., 2018; Anfodillo and Olson, 2024). In addition, empirical evidence shows that leaf area to sapwood area decreases in taller trees (McDowell et al., 2002), and that the number of sapwood rings increases with tree height (Petit et al., 2023).

Leaves should not be viewed as hydraulically independent from stems, but rather as specialized terminal organs within a continuous whole-plant hydraulic pathway, in which a substantial fraction of total resistance may reside within the leaf itself, including both xylem and extra-xylary tissues (Sack and Holbrook, 2006; Lechthaler et al., 2020). What distinguishes them mechanistically from stems is that they operate as quasi-two-dimensional, laterally leaky exchange systems, in which water is progressively redistributed across vein orders (Sack and Holbrook, 2006; Price and Enquist, 2007). Accordingly, Price and Enquist (2007) extended the WBE model to account for the distinct structural and functional organization of leaf vasculature. This broader network perspective helps explain why variation in leaf xylem architecture is unlikely to converge on a single universal scaling pattern (Price and Enquist, 2007). A fuller understanding of how leaf xylem structure shapes hydraulic function therefore requires considering conduit widening alongside with number of conduits, xylem area, and vein order within the venation hierarchy.

Although a growing body of evidence demonstrates that xylem structure within leaves can vary substantially along the longitudinal axis (**Table 1**), many recent scaling models continue to treat leaves as passive endpoints of the hydraulic network, implicitly assuming that internal leaf structure has little influence on axial scaling relationships. This discrepancy highlights a pronounced mismatch between the recognized importance of leaves in whole-plant hydraulics (Sack and Holbrook, 2006) and the limited availability of longitudinally resolved anatomical data. It is also reflected in prevailing anatomical practices, where leaf xylem traits are typically measured at a single position without reference to total leaf length or sampling location (see Gebauer, 2024). The relative lack of research on leaf xylem structural variability compared to stems may reflect a combination of methodological and conceptual constraints. Leaves constitute highly complex hydraulic systems, in which water transport occurs not only within the xylem but also across living tissues, complicating the interpretation of structure–function relationships. Additionally, the small spatial scale of leaf venation and the difficulty of obtaining precise cross-sections limit detailed anatomical analyses relative to stems. Thus, given the limited available evidence, this review does not aim to establish general rules of leaf-level tip-to-base widening, but instead focuses on synthesizing existing knowledge, identifying conceptual and methodological gaps, and promoting a methodological shift toward capturing axial variation in leaf xylem structure.

**Table 1:** Published values of the tip-to-base xylem conduit widening exponent (b) describing axial variation in leaf xylem anatomy across tree species and grasses.

Species	b	Plant taxa	Reference
*Acer pseudoplatanus*	0.41	angiosperm	Lechthaler et al., 2020
*Acer pseudoplatanus*	0.42	angiosperm	Lechthaler et al., 2019
*Cecropia obtusa*	0.549	angiosperm	Levionnois et al., 2020
*Fagus sylvatica*	0.33	angiosperm	Lechthaler et al., 2020
*Fraxinus excelsior*	0.42	angiosperm	Petit and Anfodillo, 2013
*Quercus castaneifolia*	0.55	angiosperm	Coomes et al., 2008
*Quercus cerris*	0.59	angiosperm	Coomes et al., 2008
*Quercus georgiana*	0.58	angiosperm	Coomes et al., 2008
*Quercus ilex*	0.73	angiosperm	Coomes et al., 2008
*Quercus nigra*	0.48	angiosperm	Coomes et al., 2008
*Quercus petraea*	0.42	angiosperm	Coomes et al., 2008
*Quercus rhysophylla*	0.47	angiosperm	Coomes et al., 2008
*Quercus robur*	0.48	angiosperm	Coomes et al., 2008
*Quercus rubra*	0.51	angiosperm	Coomes et al., 2008
*Quercus serrata*	0.6	angiosperm	Coomes et al., 2008
*Bromus inermis*	0.35	C3 grass	Ochteltree and Gleason, 2024
*Elymus canadensis*	0.44	C3 grass	Ochteltree and Gleason, 2024
*Andropogon gerardii*	0.41	C4 grass	Ochteltree and Gleason, 2024
*Panicum virgatum*	0.40	C4 grass	Ochteltree and Gleason, 2024
*Schizachyrium scoparium*	0.24	C4 grass	Ochteltree and Gleason, 2024
*Sorghastrum nutans*	0.26	C4 grass	Ochteltree and Gleason, 2024
*Picea abies*	0.04	conifer	Lechthaler et al., 2020
*Pinus devoniana*	0.11	conifer	Bicego et al., 2026
*Pinus montezumae*	0.13	conifer	Bicego et al., 2026
*Pinus hartwegii* (wet)	0.15	conifer	Bicego et al., 2026
*Pinus pseudostrobus*	0.13	conifer	Bicego et al., 2026
*Pinus hartwegii* (dry)	0.09	conifer	Bicego et al., 2026
*Sequoia sempervirens*	0.045	conifer	Bicego et al., 2026

Sampling design used to calculated tip-to-base xylem conduit widening exponent: sampling several points along the midrib (Petit and Anfodillo, 2013; Lechthaler et al., 2019, 2020; Ochteltree and Gleason, 2024; Bicego et al., 2026); using a petiole cross-section in combination with total leaf length (Levionnois et al., 2020); sampling three discrete points: the petiole, one-third of the way along the midrib and one-third along a secondary vein (Coomes et al., 2008).

## Leaves as an important part of whole plant hydraulic architecture

Although stems and roots account for the majority of the transport path length, a large fraction of the total resistance is concentrated near the distal components of the system, particularly within leaves (Sack and Holbrook, 2006; Lechthaler et al., 2020; Wolfe et al., 2023). Leaves differ fundamentally from stems in their geometry, function, and hydraulic organisation. Unlike stems, leaves operate as laterally leaky systems: water exits the xylem continuously along the leaf to supply mesophyll tissues and sustain transpiration through stomata (Zwieniecki et al., 2006; Ochteltree and Gleason, 2024). Leaves also experience more negative water potentials than stems (Lechthaler et al., 2020). Moreover, leaves are typically short-lived and phenologically plastic, adjusting rapidly to changing environmental conditions, whereas stems represent long-term structural investments whose anatomy integrates conditions experienced over multiple growth seasons. Together, these contrasts suggest that the selective pressures shaping leaf xylem architecture differ fundamentally from those governing stem xylem structure.

The hydraulic role of leaves cannot be fully understood without accounting for how resistance is integrated along the leaf axis. Total leaf resistance reflects the cumulative resistance encountered from the petiole or leaf base to the distal lamina and is highly sensitive to longitudinal variation in conduit diameter (Lechthaler et al., 2020; Bicego et al., 2026). Consequently, longitudinal patterns of xylem anatomy influence not only total leaf hydraulic resistance but also the spatial partitioning of water potential between vascular and mesophyll tissues.

## What do we know about tip-to-base widening in leaves?

**Figure 1:**
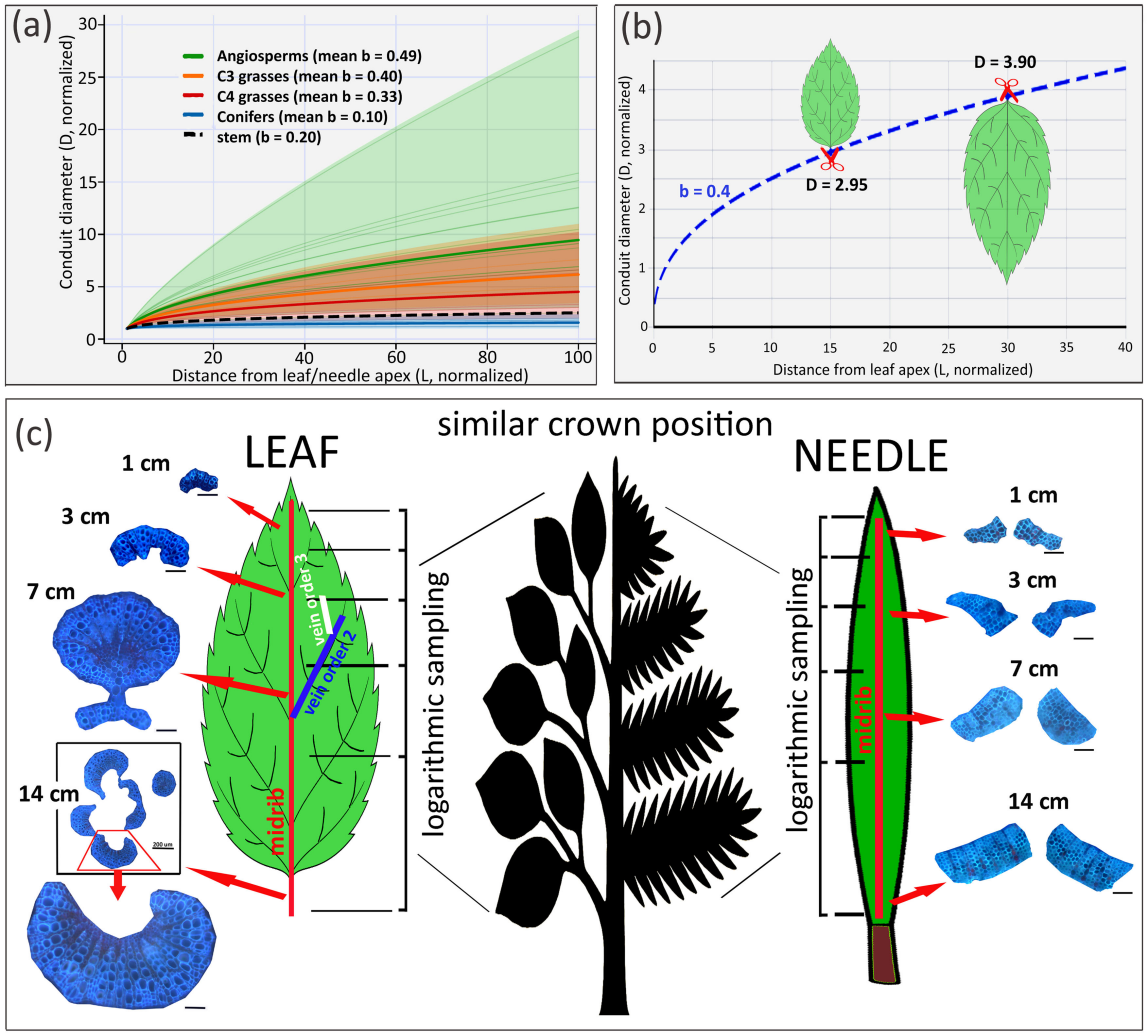
**(a)** Tip-to-base widening of xylem conduits across major plant functional groups (see **Table 1** for detail). Conduit diameter (D) is shown as a function of distance from the apex (L), following the power-law relationship D = a·L^b^ (a = 1, normalized). Thin colored lines represent individual species. Thick colored lines show group-level mean scaling for angiosperms, conifers, C3 grasses, and C4 grasses, with shaded envelopes indicating ± 95 % interspecific variability in the widening coefficient. Mean b values for each group are given in the legend. The thick black dashed line represents the observed stem-level tip-to-base widening rate (b = 0.2). Angiosperms and grasses leaves show consistently steeper scaling than conifer needles, indicating stronger tip-to-base widening along the hydraulic pathway. Small differences in tip-to-base widening translate into large, cumulative differences in hydraulic resistance, with angiosperms and grasses strongly buffering resistance accumulation along the flow path compared with conifers. **(b)** Illustration of a leaf size–related sampling artifact in anatomical analysis. Petiole cross-sections (scissor symbols) taken from two leaves differing twofold in length appear to differ in conduit diameter by 25%. However, when the tip-to-base widening of conduit diameter is accounted for (exponent b = 0.4), the leaves are anatomically equivalent. Ignoring this scaling can bias estimates of leaf hydraulic resistance, hinder comparisons among species with different leaf sizes, and misrepresent leaf size plasticity. Apparent differences may therefore reflect inconsistent or undocumented sampling positions rather than true biological variation. **(c)** Recommended sampling strategy for studying tip-to-base xylem widening in leaves and needles. At a minimum, the total length of the conductive pathway in the midrib vein (vein order 1) should be reported for each leaf or needle, together with the exact sampling position expressed as distance from the leaf tip. Sampling multiple cross-sections along the hydraulic pathway is strongly recommended, with precise positional information provided for each section. In leaves, additional cross-sections across different vein orders should also be included, with sampling positions referenced to the distal end of the conductive pathway. Logarithmic spacing of sampling points is particularly advisable where conduit dimensions change rapidly, such as near the leaf apex. In comparative studies, leaves should be sampled from similar crown position and under comparable environmental conditions. Xylem cross-sections along the midrib of *Corylus avellana* (L) leaf at distances of 1 cm, 3 cm, 7 cm, and 14 cm from the leaf tip are shown on the left. Corresponding xylem cross-sections along the midrib of *Pinus nigra* J.F. Arnold needle at the same distances from the needle tip are shown on the right. Scale bar = 50 µm. Note that the xylem cross-section of *Corylus avellana* at 14 cm from the leaf tip was too large to fit within the standard scale; therefore, it is shown separately with a scale bar of 200 µm, while only part of the cross-section is shown at a scale bar of 50 µm. Xylem cross-sections were obtained using an Olympus BX51 epifluorescence microscope equipped with a U-MWU2 filter set (Olympus Corporation, Tokyo, Japan).

A key finding emerging from these seven studies on conduit variation along the leaf axis is that the rate of tip-to-base widening of xylem conduits varies markedly among major plant taxa, with especially strong contrasts between angiosperm leaves and conifer needles (**Figure 1a**; **Table 1**). In angiosperm leaves, conduit diameter typically increases steeply from the distal lamina toward the leaf base. Reported widening exponents range from 0.2 to 0.73 (Coomes et al., 2008; Petit and Anfodillo, 2013; Lechthaler et al., 2019, 2020; Levionnois et al., 2020), with values between 0.24 and 0.44 in grasses (Ochteltree and Gleason, 2024) (**Table 1**). At these rates, basipetal widening is sufficient to nearly fully offset the increase in hydraulic resistance associated with longer conductive path lengths (Lechthaler et al., 2020; Olson et al., 2021; Bicego et al., 2026). Consequently, leaf hydraulic resistance becomes largely independent of leaf length, enabling angiosperms to evolve and sustain a wide variation in leaf size across species and environments, from tiny leaves to leaves exceeding one meter in length (Levionnois et al., 2020; Olson et al., 2021). Leaf size can therefore respond flexibly to light, temperature, and carbon economy without incurring large hydraulic costs.

In contrast, conifer needles show weak or negligible axial widening (**Figure 1a**). Reported widening exponents in pine needles range from 0.04 to 0.15 (Lechthaler et al., 2020; Bicego et al., 2026) (**Table 1**). Such low widening rates cause hydraulic resistance to increase strongly with needle length. Reducing needle length from 4 cm to 1 cm can decrease hydraulic resistance by more than 60% (Bicego et al., 2026). It should be noted that tracheids in conifer needles are much smaller than those in stems. Consequently, even if axial widening within needles is weak or absent, the total path-length resistance may still be concentrated in the needles (Lechthaler et al., 2020).

These contrasting patterns likely reflect fundamental anatomical and functional differences between angiosperm leaves and conifer needles. Conifers may not have evolved pronounced widening of needle tracheids to the same extent as angiosperms evolved vessel widening because tracheids appear to be subject to stronger functional constraints. In conifers, the same cells contribute to both water transport and mechanical support, and hydraulic safety depends strongly on pit function (Tyree and Zimmermann, 2002). Under these constraints, reducing needle length may confer a greater hydraulic benefit than increasing tracheid diameter, which could help explain why hydraulic resistance in conifer needles appears to remain closely linked to path length. Angiosperm leaves also possess complex, mostly reticulate venation networks characterized by extensive conduit branching and fusion (Sack and Holbrook, 2006). In addition to tip-to-base widening along the midrib vein, vein hierarchy introduces a secondary axis of hydraulic variation. Even in studies that do not reconstruct complete tip-to-base profiles, consistent patterns emerge across vein orders (Carvalho et al., 2017; Scoffoni et al., 2017). This hierarchical tapering concentrates hydraulic resistance in the distal portions of the leaf while maintaining highly conductive proximal pathways. The diversity of venation architectures in angiosperms likely underlies the broad range of reported conduit widening exponents. Consistent with the extended WBE framework of Price and Enquist (2007), leaf vascular scaling is not expected to follow a single universal exponent, reflecting variation in network geometry, lateral leakage, and allocation between transport and support. As a result, angiosperm leaves exhibit substantial variation in widening behavior. In contrast, conifer needles show consistently low widening exponents, likely reflecting a hydraulic strategy that prioritizes safety and structural robustness over strong compensation for path-length effects. Their comparatively simple vascular architecture, in which hydraulic resistance remains strongly dependent on the path length further constrains scaling behavior (Zwieniecki et al., 2006; Bicego et al., 2026). Both empirical and theoretical studies indicate that leaf vascular networks are shaped by multiple architectural constraints rather than a single universal design. Consequently, venation architecture provides a valuable framework for linking leaf form, hydraulic function, and scaling theory (Price and Enquist, 2007; Price et al., 2012; Bouche et al., 2014). Together these studies suggests that leaf size, xylem architecture, and whole-plant hydraulic design are tightly coordinated traits. Leaf xylem not only determines water transport efficiency at the leaf level, but also influences conduit diameters in terminal twig and, through this linkage, across the entire shoot–root system (Evans et al., 2016; Cao et al., 2022; Rivera et al., 2026).

## Sampling design to understand leaf xylem tapering

Since the pioneering work of Coomes et al. (2008), which demonstrated the functional importance of tip-to-base conduit widening in oak leaves, only six additional studies have explicitly examined longitudinal variation in leaf xylem conduit (**Table 1**). This limited attention is striking given the growing recognition of axial variation as an important determinant of hydraulic function in shoot systems (Anfodillo et al., 2006; Petit et al., 2008; Zambonini et al., 2024; Kreinert et al., 2025; Alemán-Sancheschúlz et al., 2026; Held et al., 2026). At the same time, most recent investigations of leaf xylem structure have relied on measurements from a single cross-sectional position, often without reporting the distance from the leaf tip or base (see Gebauer, 2024). While such approaches have yielded valuable insights, they provide only a partial view of leaf hydraulic organization.

If sampling positions differ among studies or are poorly documented, apparent anatomical differences may reflect methodological artifacts rather than biological variation. Treating leaves as hydraulically invariant endpoints will bias our estimate of leaf hydraulic resistance, hinder comparisons among species with different leaf sizes, and limit interpretation of leaf size plasticity under environmental stress (**Figure 1b**). Given the current state of knowledge, improving methodological transparency and sampling design therefore emerges as an important priority.

At a minimum, studies of leaf xylem anatomy should explicitly report total leaf length and the exact sampling position, expressed as distance from the leaf tip. However, I strongly recommend sampling at multiple points along the midrib, and where feasible, across vein orders (**Figure 1c**). Even relatively sparse sampling along the leaf axis can be sufficient to reveal whether, and to what extent, xylem structure varies from the leaf tip to the base. Logarithmic spacing of sampling points, as proposed by Bicego et al. (2026), is particularly effective in capturing parts where anatomical traits change most rapidly, especially near the leaf apex (**Figure 1c**). It should be also note that, in comparative studies, leaves should be sampled from similar crown position and under comparable environmental conditions (**Figure 1c**). Importantly, such approaches are technically straightforward, rely on standard anatomical methods, and can often be incorporated into existing workflows with minimal additional effort.

## Conclusions

The tip-to-base widening of xylem conduits is a well-established feature of stem hydraulic architecture, yet its occurrence and functional significance within leaves remain poorly resolved. To date, only seven studies have directly quantified axial variation in leaf conduit diameter, and just four others have examined axial variation more generally. As a result, the empirical basis for generalizing patterns of leaf-level widening remains extremely limited. Nevertheless, the available evidence clearly demonstrates that leaf xylem structure is not spatially uniform and that neglecting longitudinal variation can bias anatomical, hydraulic, and ecological interpretations. This review emphasizes the need to treat leaves as structurally heterogeneous components of the hydraulic pathway rather than as uniform terminal endpoints. At a minimum, future studies should report total leaf length and the exact sampling position along the leaf axis; however, sampling at multiple positions along the leaf axis is strongly recommended.

It must be noted that any characterization of leaf xylem that focuses on a subset of traits, whether axial variation in conduit diameter or cross-sectional variation in number of conduits and network connectivity, necessarily captures only part of a highly integrated system. From this perspective, comparisons of leaf xylem structure among individuals and species may be constrained when key dimensions variation are not considered jointly. Hence, meaningful progress will require integrative sampling strategies that capture multiple dimensions of leaf hydraulic organisation, including conduit dimensions, number of conduits, conduit length and vein network topology across the leaf axis. Such measurements should ideally be complemented by estimates of leaf size and, where feasible, by functional traits such as maximum photosynthetic rate, stomatal conductance, leaf hydraulic conductance, and embolism resistance, to better link structure with hydraulic performance.
